# Successful use of adalimumab as a conservative treatment for bilateral knee lipoma arborescens in patient with psoriatic juvenile idiopathic arthritis – case report and review of literature

**DOI:** 10.3389/fped.2022.1014536

**Published:** 2022-12-06

**Authors:** Marijan Frkovic, Magdalena Kujundzic, Mislav Cavka, Marija Jelusic

**Affiliations:** ^1^ Department of Paediatrics, University of Zagreb School of Medicine, University Hospital Centre Zagreb, Zagreb, Croatia; ^2^ Department of Diagnostic and Interventional Radiology, University of Zagreb School of Medicine, University Hospital Centre Zagreb, Zagreb, Croatia

**Keywords:** juvenile idiopathic arthritis, lipoma, magnetic resonance imaging, TNF inhibitor, adalimumab, synovectomy

## Abstract

Lipoma arborescens (LA) is a chronic, slowly progressive intra-articular mass associated with the proliferation of synovial villi. It can affect one or several joints and has been commonly described in adults with degenerative joint disease. Most patients have been diagnosed with MRI and/or biopsy findings and are usually treated with partial or total synovectomy. Case reports of LA in children, particularly with juvenile idiopathic arthritis (JIA) are scarce. We present a 16-year-old girl with a prolonged course of psoriatic JIA (initial bilateral knee affection and subsequent involvement of wrists and elbows combined with psoriatic scalp lesions) and LA of both knees. Psoriatic JIA has been diagnosed at the age of 13, with immediate start of methotrexate (MTX) therapy. Several weeks later, magnetic resonance imaging (MRI) of the right knee, performed with the aim of the most swollen joint additional evaluation, revealed synovial changes consistent with LA; arthroscopic biopsy confirmed the diagnosis. After two years of MTX treatment, despite the successful maintenance of minimal JIA activity except for repetitive bilateral knee swelling, control MRI revealed bilateral knee lesions identical to those described two years earlier in the right knee. Following the step-up approach in JIA treatment, the TNF inhibitor adalimumab was added in therapy. Finally, six months later, clinical reduction of both knees swelling was noticed with almost complete LA regression in the right, and partial regression in the left knee, confirmed by final MRI control. A conservative approach, including TNF inhibitors, instead of usually performing synovectomy, seems like a reasonable option in cases of LA with underlying JIA.

## Introduction

Lipoma arborescens (LA) is a rare, intra-articular lesion characterized by subsynovial villous proliferation of mature adipocytes (1–15). Despite its obscure etiology, it is considered a nonspecific reactive response to chronic synovial irritation (1–6, 8, 10, 15). LA is usually unilateral, with the suprapatellar pouch of the knee as the most common site of involvement (1–7, 9–11). It may also affect other joints, as well as extra-articular sites such as periarticular bursae and tendon sheaths (1, 2, 4–6, 9, 10). Slowly progressive painless swelling of the affected joint/s and episodes of joint effusion is the typical presentation (1, 2, 4, 5, 8, 9, 11, 13). MRI is the gold standard for evaluation of LA, but the definite diagnosis is usually confirmed by histological examination (1–3, 5–9, 11, 12, 14). Synovectomy is the treatment of choice and recurrences are uncommon (2, 3, 5–9, 14, 15).

Although the pathogenesis of JIA, as the most common rheumatic disease of the childhood, is closely associated with chronic synovitis, there are only several reports of LA in JIA patients (13–16). Despite the rare occurrence, LA should be considered a differential diagnosis of persistent joint/s swelling in children, adolescents, and adults in general, and in a sub-population of consistently treated JIA patients (1, 2, 5–8, 11, 14, 17).

In this paper we present a 16-year-old patient with a prolonged course of psoriatic JIA and bilateral knee LA. We also consider contemporary perspectives on the LA diagnostic algorithm, differential diagnosis and disease outcome with particular emphasis on anti-inflammatory treatment options.

## Case presentation

**Figure 1:**
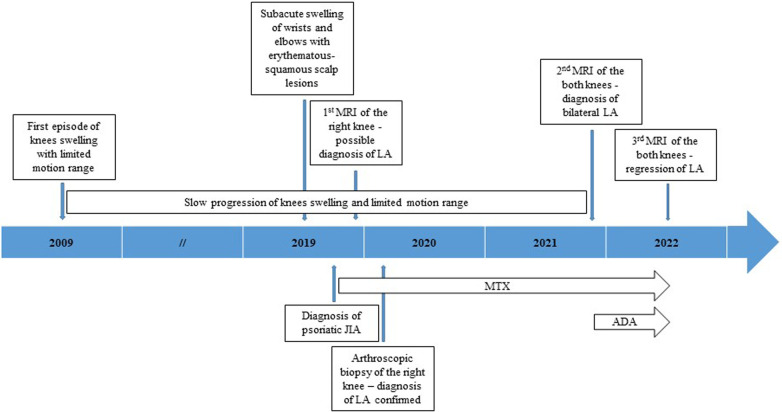
Diagram of disease progression, diagnostic evaluation, and therapeutic interventions.

A 16-year-old girl was referred to our pediatric department for the first time at the age of 13, with a 10-year history of bilateral knee swelling combined with progressive motion range reduction but without morning stiffness or mechanical symptoms, such as locking, catching, and giving way and subacute development of symmetrical elbow and wrist swelling with prolonged morning stiffness along with multiple erythematous-squamous scalp lesions for the past few months (Figure 1).

The first physical examination demonstrated significant, diffuse, painless swelling of both knees (more prominent right) with reduced flexion up to 30 degrees, and less extensive, painless, symmetrical elbow and wrist swelling without limited range of motion. Several erythematous-squamous lesions, 1 cm to 3 cm in diameter, were detected on the scalp with no hair loss.

Laboratory findings were unremarkable other than low-positive ANA titer. x-ray and US evaluation of the clinically affected joints showed para-articular osteopenia with irregularly shaped epiphyseal ossification centers and synovial hypertrophy with joint effusion, respectively. Diagnostic needle aspiration of the right knee was performed, and 15-ml yellowish, jelly-like material with cytological signs of chronic inflammation was obtained.

According to the results of the diagnostic evaluation, a diagnosis of psoriatic JIA has been established.

**Figure 2:**
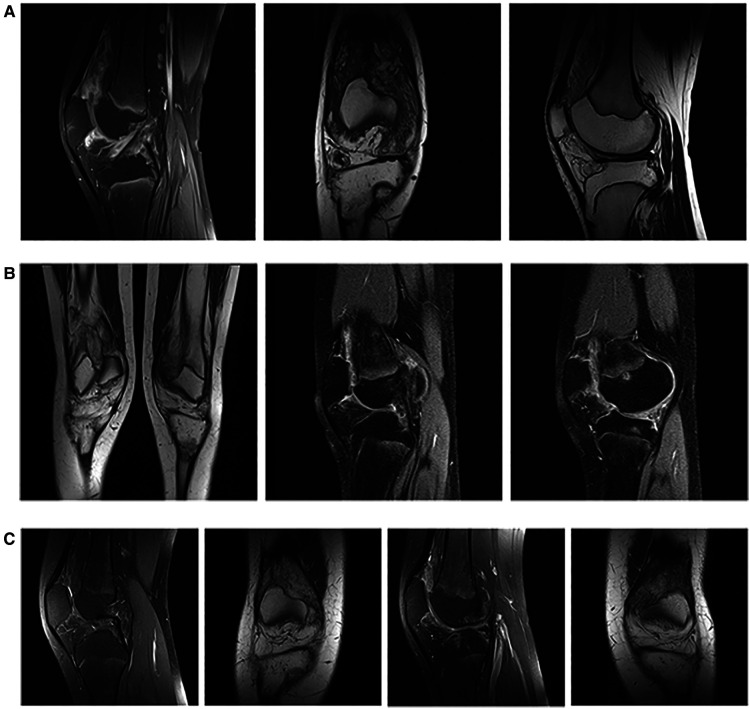
Imaging examinations. (**A**) Initial MRI scanning of the right knee [MRI Proton Density with Fat Saturation (PDFS), sagittal plane; MRI T1 weighted image, coronal plane; MRI T1 weighted image, sagittal plane] showed the extended intra-articular synovial proliferation with fat inclusions combined with multiple marginal bony erosion of the proximal tibia, both femoral condyles and patella along with altered morphology of both menisci. (**B**) Control MRI of both knees (MRI T1 weighted image, coronal plane; MRI Proton Density with Fat Saturation (PDFS), sagittal plane, right; MRI Proton Density with Fat Saturation (PDFS), sagittal plane, left) performed after 2 years of MTX therapy with almost the same, bilateral lesions as one on the initial MRI of the right knee. (**C**) Second MRI control of both knees [MRI Proton Density with Fat Saturation (PDFS), sagittal/coronal plane, right/left] with almost complete regression of the synovial proliferation in the right, and partial regression in the left knee after 6 months of TNF inhibitor therapy.

Beside the knee intra-articular steroid injection, treatment included subcutaneous methotrexate (MTX) 15 mg/m^2^ (20 mg) weekly and intensive physiotherapy. A complete clinical resolution of all affected joints followed the therapy introduction within several weeks, except for the persistent swelling of the knees. Diagnostic evaluation was completed with the contrast enhancement MRI of the more prominent swollen right knee. It depicted extended intra-articular synovial proliferation with fat inclusions, multiple marginal bony erosion of the proximal tibia, both femoral condyles and patella along with altered morphology of both menisci (Figure 2A). A diagnosis of chronic synovitis with LA was considered. Histological analysis of the specimens obtained by arthroscopic biopsy revealed hypertrophic synovium covered with 2–3 lines of synovial cells and stromal lobules of mature adipocytes with focal inflammatory infiltration—finding consistent with LA and underlying chronic synovitis.

Considering the young age of our patient and the diagnosis of psoriatic JIA, as a chronic, inflammatory underlying disease, conservative and expectative approach concerning the LA was preferred. The patient received the prescribed therapy regularly but was lost from the regular follow-up for the next year and the half. Fortunately, minimal disease activity of psoriatic JIA was successfully maintained during the specified period, without signs of arthritis of the affected joints, except the fluctuating bilateral knee swelling with reduced flexion up to 20 degrees. Control MRI of both knees at the age of 15 revealed almost the same, bilateral lesions similar to one described on the initial MRI of the right knee (Figure 2B).

Following the step-up approach of JIA treatment, TNF inhibitor—adalimumab was finally introduced. Within the next 6 months, bilateral knee swelling, and reduced flexion improved with almost complete LA regression in the right, and partial regression in the left knee, confirmed by final MRI control (Figure 2C). At the last visit the patient is satisfied with the general improvement of her condition, able to regularly conduct her daily activities with restrictions in terms of avoiding physical overloads.

## Discussion

Although the etiology and pathogenesis of LA has not yet been well defined, according to most authors, it is primarily related to chronic synovial irritation, particularly in cases of osteoarthritis or previous trauma as the triggering factors (1–7, 15). During the last decade a growing number of reports advert the inflammatory diseases, such as rheumatoid arthritis, psoriatic arthritis or psoriasis, autoimmune uveitis and different types of JIA, as possible underlying conditions related to the development of LA (2, 4–6, 10, 14, 15). Simultaneously, most authors are doubtful about the success of anti-inflammatory therapy related to LA in patients with inflammatory diseases, and frequently recommend synovectomy as a standardly used, definitive treatment (12–15).

LA has rather low incidence, and up till now around only 200 cases have been reported in adults and children (3). The incidence of LA among children and adolescents, particularly with inflammatory diseases, is extremely low (10, 11). A couple of dozen childhood cases have been reported to date; however, all authors have described only one or two patients for each study (7–18). Among these, there are only a few explicit cases of commonly unsuccessful conservative therapy of LA in children with JIA (11–16) (Table 1). Cil et al. and Bouayed et al. reported two similar cases of 13-year-old girls with bilateral knee LA initially diagnosed with oligoarticular JIA and unsuccessfully treated with an unspecified dose of MTX for 8 years and with MTX 25 mg weekly for year and a half, respectively (11, 12). Zeybek et al. presented a case of partially successful MTX treatment in a 17-year-old girl with a 5-year history of JIA and bilateral knee LA. The girl was finally scheduled for arthroscopic synovectomy (13). Xue et al. reported a 16-year-old girl with a 4-year history of juvenile spondyloarthritis and bilateral knee LA diagnosed during the treatment with TNF inhibitor etanercept 25 mg weekly, subsequently coupled with MTX 10 mg weekly (14). Batu et al. described an 11-year-old boy with a 4-year history of psoriatic arthritis initially treated with an unspecified dose of MTX weekly and coupled with also unspecified dose of etanercept weekly a year later due to the disease flare. Bilateral knee LA was discovered after subacute knees swelling during the therapy course (15).

**Table 1:** Summary of lipoma arborescens reported cases in children with juvenile idiopathic arthritis.

Author (year)	Age, sex	Diagnostic procedures	Comorbidities	Localization of LA	Initial (conservative) therapy	Surgical therapy	Outcome
Cil A et al. (2005)	13, F	MRI, biopsy	JIA	Knees	MTX, NSAID	Arthroscopic synovectomy	Complete resolution
Bouayed K et al. (2017)	13, F	MRI, arthroscopic biopsy	JIA	Knees	NSAID, MTX	–	Partial relief from symptoms
Zeybek GE et al. (2019)	17, F	MRI	JIA	Knees	sulfasalazine, intra-articular glucocorticoid, MTX	Arthroscopic synovectomy in perspective	Partial relief from symptoms
Xue J et al. (2013)	16, F	MRI, arthroscopic biopsy	JIA—jSpA	Knees	sulfasalazine, MTX, etanercept	Arthroscopic synovectomy	Complete resolution
Batu ED et al. (2020)	11, M	MRI	Psoriatic JIA	Knees	NSAID, MTX, etanercept, intra-articular glucocorticoid	Arthroscopic synovectomy	Complete resolution
Dail CS et al. (2020)	16, M	MRI, biopsy	JIA	Right knee and elbow	naproxen, MTX	Partial synovectomy of the right knee	Partial relief from symptoms

MRI, magnetic resonance imaging; JIA, juvenile idiopathic arthritis; jSpA, juvenile spondyloarthritis; LA, lipoma arborescens; NSAID, nonsteroidal anti-inflammatory drugs; MTX, methotrexate.

MRI in JIA is recommended during the diagnostic work-up and/or, over the disease course, in case of any doubt about an alternative diagnosis (1, 2, 5, 6, 9, 10, 12, 14). In the case of LA, MRI findings usually allow accurate identification as well as evaluation of size and grade. LA typically presents as frond-like areas of proliferation that have signal characteristics isointense with subcutaneous fat. The hypertrophied subsynovial fatty tissue does not enhance while the overlying synovium often shows diffuse enhancement after intravenous contrast administration (1, 4, 5, 14–17). Besides the typical characteristics of LA, the adipocytes may incompletely replace the synovial tissue, so atypical MRI findings with dominant synovial proliferation or irregular mixture of synovial and fatty tissue may also be observed (1, 14). Because of the possible atypical LA presentation and other differential diagnoses, histopathological examination of the specimen obtained by needle or arthroscopic biopsy is usually recommended as a confirmative diagnostic procedure (2, 6, 14, 17). In all above-mentioned cases of unsuccessful conservative therapy of the underlying JIA, LA was confirmed by the combination of MRI and histopathological analysis. In majority of reports of LA in children, including those with unsuccessful anti-inflammatory therapy, symptoms were resolved after arthroscopic synovectomy (Table 1).

Our patient was almost simultaneously diagnosed with psoriatic JIA and initially right knee LA, suspected by MRI and confirmed by arthroscopic biopsy, with immediate start of MTX 20 weekly as a standard therapy of psoriatic JIA. Two years later, MRI performed due to the persistent bilateral knee swelling, revealed symmetrical LA. Additional therapy with the TNF inhibitor adalimumab, 40 mg weekly during the next 6-month period resulted in clinical and MRI regression of both knees swelling and LA lesions, respectively.

Since this was the first case of LA in the 40-year history of our department (among 80–100 newly diagnosed cases of JIA per year), and due to unspecific description of initial MRI, we used the conventional diagnostic approach and confirmed the diagnosis of LA by arthroscopic biopsy. Some recent papers propose only MRI evaluation in cases of typical LA imaging, to avoid the biopsy as an invasive diagnostic procedure (1, 2, 5, 8, 11, 14).

We based our treatment approach on the commonly accepted opinion on the secondary development of the LA on the possible basis of the underlying inflammatory diseases (in our case psoriatic JIA) which are otherwise successfully treated with anti-inflammatory drugs, including TNF inhibitors. Additionally, Fraser et al. demonstrated that LA tissue itself might release TNF, which contributes to joint inflammation and therefore could theoretically be also susceptible to the action of TNF inhibitors (19).

Except our case, the only similar report of successful anti-inflammatory therapy in an adolescent with LA and underlying JIA was presented by Dail et al. They described a 16-year-old boy with LA of the right knee and right elbow combined with elevated levels of inflammatory markers, suggesting underlying inflammatory synovitis, in particular JIA. MTX was prescribed with satisfying therapy effects within several weeks. The same success was repeated after restarting the same therapy due to the loss to follow-up for a one year (16) (Table 1).

As opposite prospective, based on the review of literature included in their report, Xue et al. concluded that LA cannot be improved by anti-inflammatory therapy and recommended the arthroscopic synovectomy as the exclusive management of LA (14). Reports of Xue et al. and Batu et al. even indicated the possibility of developing LA during TNF inhibitor therapy (etanercept) of JIA (14, 15).

In our opinion, all options of consistent anti-inflammatory therapy with correct doses of prescribed medications (including “intensive protocols” and “switching/cycling” of biologics), primarily directed toward the treatment of underlying inflammatory disease, should be considered before definitive decision on LA surgical procedures. Slow progression of this benign lesion leaves plenty of time for tailoring different conservative options (1).

## Conclusion

Here, we present the first case of successful use of the TNF inhibitor adalimumab for treating bilateral knee LA in a patient with psoriatic JIA. Although rare, particularly in pediatric and adolescent patients, LA must be considered in the differential diagnosis of chronic joint swelling. In cases of JIA as a possible underlying condition of LA in children, all anti-inflammatory treatment options, including TNF inhibitor therapy, should be considered before synovectomy, as a procedure generally reserved for types of primary or extended secondary lesions.

## Data Availability

The raw data supporting the conclusions of this article will be made available by the authors, without undue reservation.

## References

[1] Sanamandra SK Ong KO. Lipoma arborescens. Singapore Med J. (2014) 55:5–10; quiz 11. 10.11622/smedj.2014003 24452971 PMC4291913

[2] Tsifountoudis I Kapoutsis D Tzavellas AN Kalaitzoglou I Tsikes A Gkouvas G. Lipoma Arborescens of the knee: report of three cases and review of the literature. Case Rep Med. (2017) 2017:3569512. 10.1155/2017/3569512 28243256 PMC5294362

[3] Wang CK Alfayez S Marwan Y Martineau PA Burman M. Knee arthroscopy for the treatment of lipoma arborescens: a systematic review of the literature. JBJS Rev. (2019) 7(4):e8. 10.2106/JBJS.RVW.18.00139 31021893

[4] Howe BM Wenger DE. Lipoma arborescens: comparison of typical and atypical disease presentations. Clin Radiol. (2013) 68:1220–6. 10.1016/j.crad.2013.07.002 23969149

[5] De Vleeschhouwer M Van Den Steen E Vanderstraeten G Huysse W De Neve J Vanden Bossche L. Lipoma Arborescens: review of an uncommon cause for swelling of the knee. Case Rep Orthop. (2016) 2016:9538075. 10.1155/2016/9538075 27293937 PMC4884798

[6] Bent MA Varacallo M Fox EJ Voss S Frauenhoffer EE. Lipoma arborescens and coexisting psoriatic arthritis: a case report and review of the literature. JBJS Case Connect. (2013) 3(4):e121. 10.2106/JBJS.CC.M.00079 29252521

[7] Baidoo PK Nketiah-Boakye F Tano EK Al-Hassan M Yorke GOM Awoonor-Williams R Lipoma arborescens in a 16-year-old male: a case report. Clin Case Rep. (2021) 9:e05230. 10.1002/ccr3.5230 34976396 PMC8684577

[8] Aydin G Keleş I Karagülle Kendi AT Yilmaz LÖ Orkun S. Case report: lipoma Arborescens in childhood: a report of two sisters. Arch Rheumatol. (2012) 27:56–62. 10.5606/tjr.2012.007

[9] Nevins LCE Tenfelde AM. Lipoma Arborescens in a 10-year-old boy. J Am Acad Orthop Surg Glob Res Rev. (2020) 4:e20.00108. 10.5435/JAAOSGlobal-D-20-00108 33355429

[10] Sharma SD Bagri DR Gupta RK Sarna A. Lipoma arborescens. Indian J Pediatr. (2011) 78:1016–8. 10.1007/s12098-011-0372-6 21359501

[11] Cil A Atay OA Aydingöz U Tetik O Gedikoğlu G Doral MN. Bilateral lipoma arborescens of the knee in a child: a case report. Knee Surg Sports Traumatol Arthrosc. (2005) 13:463–7. 10.1007/s00167-004-0538-0 16170581

[12] Bouayed K Cherqaoui A Salam S Karkouri M Mikou N. Lipoma arborescens: a rare cause of bilateral pseudo-arthritis of the knee in children. Joint Bone Spine. (2017) 84:639–40. 10.1016/j.jbspin.2016.11.003 28185767

[13] Zeybek GE Kalin S Sozeri B. Progressive bilateral lipoma arborescens of the knee caused by uncontrolled juvenile idiopathic arthritis. North Clin Istanb. (2019) 7:512–5. 10.14744/nci.2019.24471 33163890 PMC7603851

[14] Xue J Alario AJ Nelson SD Wu H. Progressive bilateral lipoma arborescens of the knee complicated by juvenile spondyloarthropathy: a case report and review of the literature. Semin Arthritis Rheum. (2013) 43:259–63. 10.1016/j.semarthrit.2012.12.022 23352250

[15] Batu ED Sonmez HE Kösemehmetoğlu K Özer H Aydingöz Ü. Lipoma arborescens associated with psoriatic arthritis in an adolescent boy: a case report and review of the literature. J Clin Rheumatol. (2020) 26:e47–49. 10.1097/RHU.0000000000000833 32073533

[16] Dail CS Dietz KR Murati MA Correll CK. Teenaged boy with lipoma Arborescens of the knee and elbow and presumed juvenile idiopathic arthritis. Arthritis Rheumatol. (2020) 72:315. 10.1002/art.41167 31729184

[17] Mistovich RJ O'Toole PO Chauvin NA Wilkins BJ Ganley TJ. Left knee pain and bilateral knee swelling in an adolescent. Clin Orthop Relat Res. (2014) 472:3591–8. 10.1007/s11999-014-3883-4 25163688 PMC4182385

[18] Senocak E Gurel K Gurel S Ozturan KE Cakici H Yilmaz F Lipoma arborescens of the suprapatellar Bursa and extensor digitorum longus tendon sheath: report of 2 cases. J Ultrasound Med. (2007) 26:1427–33. 10.7863/jum.2007.26.10.1427 17901145

[19] Fraser AR Perry ME Crilly A Reilly JH Hueber AJ McInnes IB. Lipoma arborescens co-existing with psoriatic arthritis releases tumour necrosis factor alpha and matrix metalloproteinase 3. Ann Rheum Dis. (2010) 69:776–7. 10.1136/ard.2008.106047 20237127

